# Establishment of a Replicating Plasmid in *Rickettsia prowazekii*

**DOI:** 10.1371/journal.pone.0034715

**Published:** 2012-04-17

**Authors:** David O. Wood, Andria Hines, Aimee M. Tucker, Andrew Woodard, Lonnie O. Driskell, Nicole Y. Burkhardt, Timothy J. Kurtti, Gerald D. Baldridge, Ulrike G. Munderloh

**Affiliations:** 1 Department of Microbiology and Immunology, Laboratory of Molecular Biology, University of South Alabama College of Medicine, Mobile, Alabama, United States of America; 2 Department of Entomology, University of Minnesota, St. Paul, Minnesota, United States of America; Louisiana State University and A & M College, United States of America

## Abstract

*Rickettsia prowazekii*, the causative agent of epidemic typhus, grows only within the cytosol of eukaryotic host cells. This obligate intracellular lifestyle has restricted the genetic analysis of this pathogen and critical tools, such as replicating plasmid vectors, have not been developed for this species. Although replicating plasmids have not been reported in *R. prowazekii*, the existence of well-characterized plasmids in several less pathogenic rickettsial species provides an opportunity to expand the genetic systems available for the study of this human pathogen. Competent *R. prowazekii* were transformed with pRAM18dRGA, a 10.3 kb vector derived from pRAM18 of *R. amblyommii*. A plasmid-containing population of *R. prowazekii* was obtained following growth under antibiotic selection, and the rickettsial plasmid was maintained extrachromosomally throughout multiple passages. The transformant population exhibited a generation time comparable to that of the wild type strain with a copy number of approximately 1 plasmid per rickettsia. These results demonstrate for the first time that a plasmid can be maintained in *R. prowazekii*, providing an important genetic tool for the study of this obligate intracellular pathogen.

## Introduction

*Rickettsia prowazekii*, the causative agent of epidemic typhus and a Category B Select Agent, is an obligate intracellular bacterium that grows directly within the cytosol of eukaryotic host cells. The obligate intracellular nature of *R. prowazekii* growth places considerable restrictions on the genetic manipulation of this pathogen. Foremost, it is essential that manipulations do not prevent the rickettsiae from infecting host cells, a requirement for rickettsial survival and growth. Since *R. prowazekii* cannot be grown as colonies on an agar surface using axenic media, standard bacterial cloning protocols are unavailable. In addition, plaquing techniques currently used for cloning spotted fever group rickettsiae that can polymerize actin for intracellular movement are problematic for *R. prowazekii*, which is deficient in actin polymerization [1], [2], [3]. Thus, clonal populations of *R. prowazekii* mutants must be isolated using labor-intensive and time-consuming techniques such as limiting dilution [4], [5], [6], [7]. This inability to form colonies or efficiently form plaques prohibits the precise determination of *R. prowazekii* transformation frequencies [8]. However, despite these barriers, advances in the genetic manipulation of this intractable organism have been made. For example, identification of antibiotics suitable for the selection of rickettsial transformants [4], [5], the use of fluorescent proteins as reporter genes [9], the adaptation of transposon systems for generating *R. prowazekii* insertional mutants [5], [6], and the directed knockout of a rickettsial gene [7] have now been reported. Although complementation of an *R. rickettsii* gene mutation using the *Himar1* transposon system was recently achieved [8], the *R. prowazekii* genetic toolbox still lacks a replicating plasmid for extrachromosomal gene expression studies that would not result in chromosomal disruption. Fortunately, the demonstration that some rickettsial species harbor plasmids has added another genetic component to the rickettsial gene repertoire.

Originally, the first rickettsial genome sequencing projects targeting rickettsial pathogens failed to find plasmids, supporting the hypothesis that rickettsial species did not contain extrachromosomal elements. However, beginning with the identification of plasmids in *R. felis* [10], followed by plasmid identifications in rickettsiae ranging from other pathogens (e.g. *R. akari*) to arthropod endosymbionts (e.g. *R. amblyommii*, *R. bellii*, *R rhipicephali*, and the rickettsial endosymbionts of *Ixodes scapularis*, REIS) [11], [12], [13], it became evident that plasmids are not uncommon in rickettsial species. However, after multiple sequencing projects examining different strains, plasmids still have not been identified in *R. prowazekii*. To evaluate whether *R. prowazekii* can maintain a plasmid and to generate an additional tool for the genetic analysis of this pathogen, we introduced a recombinant plasmid derived from one of the natural plasmids of *R. amblyommii* into *R. prowazekii* and characterized its maintenance and its effect on rickettsial growth. To our knowledge this is the first plasmid shown to stably replicate in *R. prowazekii*, opening the door to genetic analyses requiring an extrachromosomal platform.

## Materials and Methods

### Bacterial strains and culture conditions

*R. prowazekii* Madrid E strain rickettsiae (Passage 283) were propagated and purified from hen egg yolk sacs [14] and L929 mouse fibroblasts (American Type Culture Collection, Manassas, VA, ATCC Number CCL-1) as described previously [5]. Purified rickettsiae were stored frozen in a sucrose-phosphate-glutamate-magnesium solution (0.218 M sucrose, 3.76 mM KH_2_PO_4_, 7.1 mM K_2_HPO_4_, 4.9 mM potassium glutamate, and 10 mM MgCl_2_). Rickettsiae-infected L929 cells were grown in modified Eagle medium (Mediatech, Inc., Herndon, VA) supplemented with 10% newborn calf serum (Hyclone, Logan, UT) and 2 mM L-glutamine (Mediatech) in an atmosphere of 5% CO_2_ at 34°C. *Escherichia coli* strain XL1-Blue (Stratagene, La Jolla, CA) was used as a recipient for shuttle vector pRAM18dRGA [15] and for preparation of plasmid DNA used in rickettsial transformation. XL1-Blue was cultured in Luria-Bertani (LB) medium at 37°C. For selection of *E. coli* transformants, rifampin was added to a final concentration of 50 µg/ml.

### *R. prowazekii* transformation

Purified rickettsiae were made competent for transformation and electroporated, as previously described [5], [16], in the presence of 14 µg of pRAM18dRGA plasmid DNA. Twenty-four hours following electroporation and infection of mouse fibroblast L929 cells, rifampin was added to a final concentration of 200 ng/ml and rifampin selection was maintained throughout the experiment. The introduction of a gene conferring rifampin resistance into *R. prowazekii* has been approved by both the University of South Alabama Institutional Biosafety Committee and the Centers for Disease Control, Division of Select Agents and Toxins. Rickettsial infection and growth was monitored by microscopic examination of Gimenez-stained [17] infected cells on cover slips. For infection levels and calculations of rickettsiae per cell, 100 cells were analyzed at each time point. Fluorescent images were acquired using a Nikon Eclipse T2000-U fluorescent microscope and images overlaid using MetaMorph Imaging System software (Universal Imaging Corporation).

### Plasmid recovery

Total DNA from the rifampin-resistant rickettsial population (designated ME-pRAM18dRGA) grown in L929 cells was extracted using the DNEasy Blood & Tissue Kit (Qiagen, Valencia, CA). Following total DNA extraction, plasmid DNA was isolated using the Qiagen Plasmid Mini kit. Plasmid DNA (200 ng) was electroporated into XL-1 Blue electrocompetent *E. coli* and transformants selected on LB medium agar plates containing 50 µg/ml rifampin. Resistant colonies were amplified, plasmid DNA extracted and subsequently sequenced by primer walking at the Iowa State University DNA Facility.

### Rickettsial growth analyses

To compare growth characteristics of the ME-pRAM18dRGA to that of the parent Madrid E strain, L929 cells were infected in suspension for 1 hour with either ME-pRAM18dRGA or the wild type Madrid E strain at similar multiplicities of infection. The infected cells were seeded in 60 mm dishes. Samples from each infection were harvested approximately every 24 hours. DNA was extracted from approximately 1×10^6^ infected cells using the Archive Pure DNA Cell/Tissue Kit (5 Prime Inc., Gaithersburg, MD). At sampling times, the medium was removed and adherent cells were gently rinsed with phosphate buffered saline (PBS). Cells were immediately lysed in the dish using 800 µl of Archive Pure lysis solution and total DNA (L929 and rickettsiae) was extracted. DNA concentrations were determined using the NanoDrop 1000 spectrophotometer (Thermo Scientific, Wilmington, DE) and aliquots were diluted to a final working concentration of 10 ng/µl in RT-PCR water (Applied Biosystems, Austin, TX). Samples were prepared from master mixes, so that each reaction contained 10 ng of DNA, and analyzed by quantitative PCR (QPCR). Bacterial, host, and plasmid genome equivalents were determined by targeting the single-copy *R. prowazekii rho* (RP521) chromosomal gene, the host β-actin gene, and the *gfp_uv_* gene of pRAM18dRGA. Primer pairs specific for each gene are listed in Table 1. Prior to QPCR analyses, primer pair specificity was validated for each target at the working dilution using either rickettsial genomic DNA, L929 cell genomic DNA, or the pRAM18dRGA plasmid. Assays were performed using the LightCycler® DNA Master SYBR Green I QPCR master mix (Roche, Mannheim, Germany), 500 nM concentration of each primer, and a Cepheid Smart Cycler® according to the manufacturer's protocol (Cepheid, Sunnyvale, CA). Cycle parameters were 1 cycle at 95°C for 2 min, followed by 40 cycles at 95°C for 15 s, 52.3°C (*rho*), 54°C (actin) or 58°C (*gfp_uv_*) for 15 s, and 72°C for 15 s. Amplification specificity was confirmed by melting-curve analysis. Data acquisition and analysis were performed using the Cepheid Smart Cycler Version 2.0c software. Genome equivalents were determined by comparison to standard curves. Three independent biological samples were analyzed in duplicate for each time point.

**Table 1:** Primers pairs.

Gene	Strand	Sequence	Size (bp)	Efficiency^a^
Actin	F	CCCTACAGTGCTGTGGGTTT	178	93%
	R	GACATGCAAGGAGTGCAAGA		
Rho	F	CCTGCAAGTAGACATGTGC	179	95%
	R	AGTGCATTAGCATCAACACC		
GFPuv	F	CTTTTCGTTGGGATCTTTCG	119	104%
	R	ACATCACGGCAGACAAACAA		
GFP probe	F	ATGAGTAAAGGAGAAGAACTTTTCAC	716	
	R	TTATTTGTAGAGCTCATCCATGCC		

a Efficiency = 10∧(−1/slope of the standard curve)−1)*100.

### Southern blot analyses

Total DNA was isolated from rickettsiae grown in L929 cells using the DNeasy Blood and Tissue Kit (Qiagen). DNA (2 µg) was digested with SpeI, HindIII, or XhoI and the resulting DNA fragments separated by agarose gel electrophoresis. DNA was subsequently transferred to Nytran® SuPerCharge membranes (Schleicher & Schuell, Keene, NH). A *gfp_uv_*-specific probe used in Southern hybridizations [18] was PCR amplified (Table 1) and labeled using [α-^32^P]dATP (MP Biomedicals, Inc., Philadelphia, PA) and the Multiprime DNA labeling system (Amersham, Piscataway, NJ). Hybridized fragments were visualized using a Cyclone Plus phosphoimager (Perkin Elmer, Waltham, MA).

## Results

### Plasmid maintenance in *R. prowazekii*

**Figure 1:**
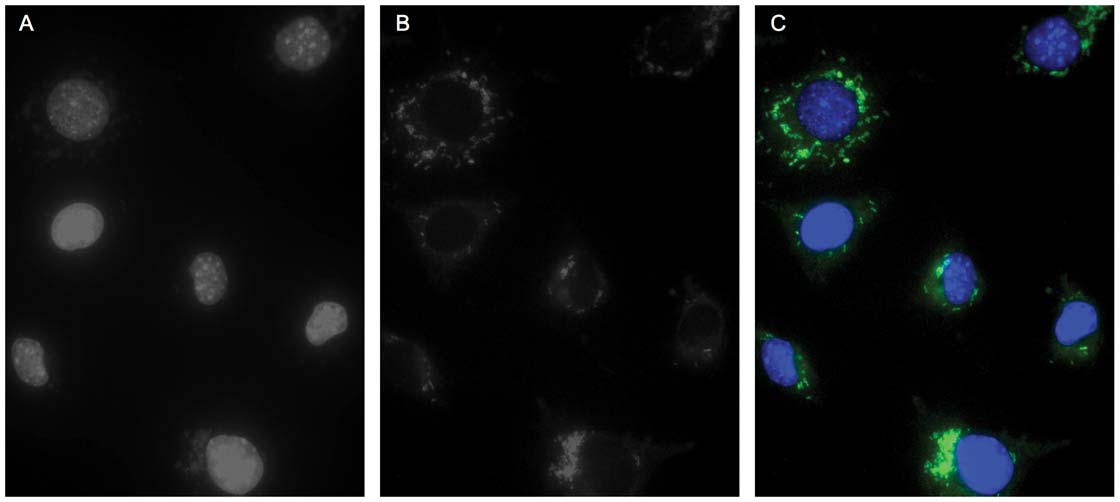
Fluorescent imaging of ME-pRAM18dRGA growing in L929 fibroblasts. L929 fibroblasts infected with ME-pRAM18dRGA were analyzed for the expression of GFP_uv_. (A) Eukaryotic nuclei and rickettsiae are visualized using DNA counter-stain DAPI. (B) Fluorescent rickettsiae expressing GFP_UV_. (C) A single plane overlay of images in Panels A and B showing that each infected cell contains fluorescent transformed rickettsiae.

**Figure 2:**
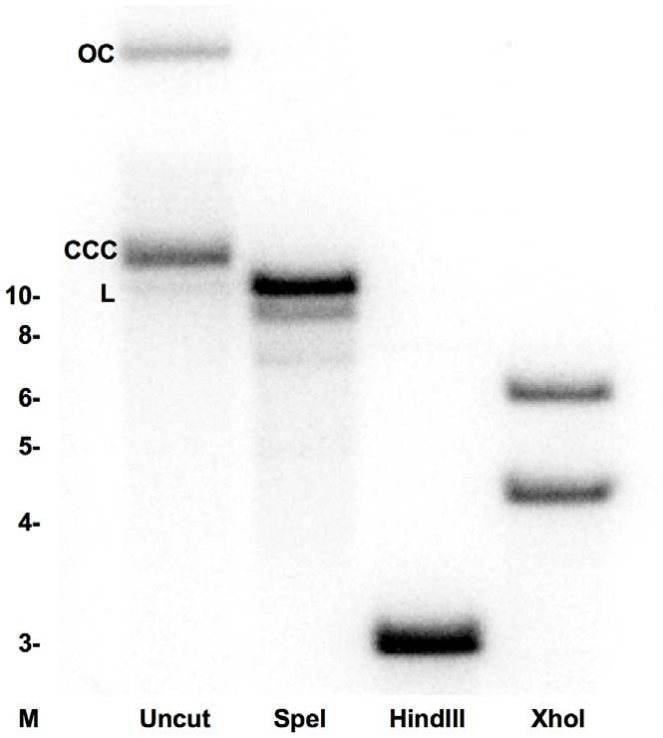
Southern blot analysis of DNA extracted from ME-pRAM18dRGA using a probe specific for *gfp_UV_*. Lanes are labeled as uncut or by the enzyme used in the digestion. Locations of molecular size markers (in kB) are indicated on the left. Plasmid forms in the uncut lane are L- linear, CCC- closed circular, and OC- open circular.

Plasmid pRAM18dRGA was introduced into competent *R. prowazekii* Madrid E rickettsiae via electroporation. Selection with rifampin was initiated 24 hours following electroporation. On day 11, a sample of the rickettsiae-infected cells was harvested, DNA extracted and examined by PCR for the presence of a rickettsial chromosomal gene (*sdh*) and for two genes (*arr2_RP_* and *gfp_uv_*) contained on the plasmid. A predicted PCR product was obtained for each gene (data not shown), indicating the presence of plasmid DNA within the rifampin resistant population. Continued expansion of the infected host cell population generated a slowly increasing population of rifampin-resistant rickettsiae that exhibited fluorescence when examined microscopically (Fig. 1). Rickettsiae were isolated from this population and used to infect L929 cells at a high multiplicity of infection to increase the percentage of infected cells. The resulting rifampin-resistant rickettsial population (designated ME-pRAM18dRGA) was subsequently analyzed for the presence of plasmid DNA by Southern hybridization (Fig. 2) using a probe that spans the coding region of *gfp_uv_*. The three physical forms of plasmid DNA (linear, covalently closed circular, and open circular) can be observed in the Uncut lane of Figure 2, demonstrating the extrachromosomal nature of pRAM18dRGA in the ME-pRAM18dRGA strain. This is supported by the presence of predicted restriction patterns for SpeI (linearizes the 10 kb plasmid), HindIII (generates a 2854 bp fragment containing the *gfp_uv_* gene) and XhoI (cuts within the *gfp_uv_* gene generating two labeled fragments of 4263 bp and 5985 bp). The two faint bands that appear below the linear fragment in the SpeI digested DNA are likely the result of DNA degradation. No hybridization to *R. prowazekii* chromosomal DNA or to L929 cell DNA was observed (data not shown). The absence of additional bands on the Southern blot supports the data that the plasmid has remained extrachromosomal and has not incorporated into the rickettsial chromosome at a detectable level.

The ME-pRAM18dRGA population was serially passaged more than 10 times for a period of greater than 30 days, under selection, without the loss of fluorescence. For serial passages, confluent cell monolayers were trypsin-treated and cells harvested and seeded into new flasks at a 1∶3 dilution. Cells reached confluence after approximately three days of growth. After extensive passaging, an extrachromosomal plasmid, with the predicted restriction pattern, could still be isolated from the rickettsiae. In addition, pRAM18dRGA could still be isolated from rickettsiae harvested from L929 cells that were serially passaged in the absence of selective pressure (rifampin) for more than two weeks.

### The effect of plasmid maintenance on rickettsial growth

**Figure 3:**
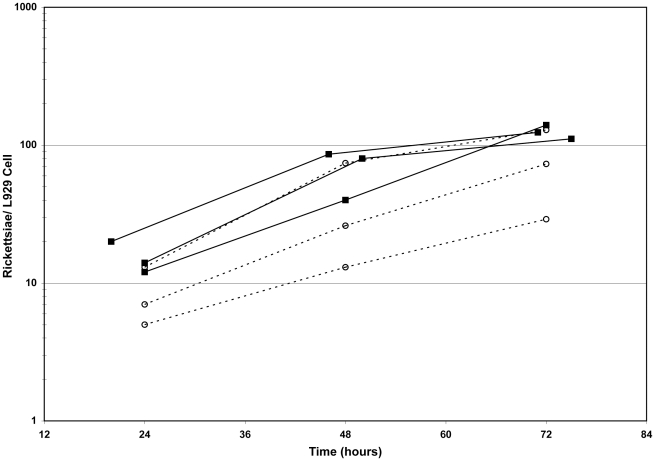
Growth curves for Madrid E (- - - -) and ME-pRAM18dRGA(—). Rickettsial growth in cultures of adherent L929 cells was followed by determining genome equivalents of an *R. prowazekii* chromosomal gene (*rho*) and the host cell actin gene. Growth is expressed as the number of rickettsiae per host cell. Each line represents an independent experiment.

Growth of ME-pRAM18dRGA was evaluated by determination of rickettsial genome equivalents per host cell using QPCR and primers (Table 1) specific for the *R. prowazekii rho* gene and the host cell actin gene. Infections were initiated with rickettsiae harvested from L929 cells, and for each infection, the percent of infected L929 cells was greater than 90%. Evaluating the increasing number of rickettsiae per adherent cell permitted a comparison of the two strains (Madrid E and ME-pRAM18dRGA) (Fig. 3). Growth of the two rickettsial strains was comparable with each exhibiting a generation time of approximately 13 and 12 hours respectively.

While the Southern blot and QPCR data cannot eliminate the possibility that spontaneous rifampin-resistant rickettsiae lacking a plasmid exist in the population, plasmid copy number (see next section) did not change appreciably over the time course of the growth curve suggesting that such a background population did not significantly affect the growth analysis. In addition, examination of cells infected with a population of plasmid transformed *R. prowazekii* revealed that every infected cell contained fluorescent rickettsiae (Fig. 1). Absolute confirmation will require the isolation and expansion of a single rickettsia by limiting dilution.

### Plasmid copy number

**Figure 4:**
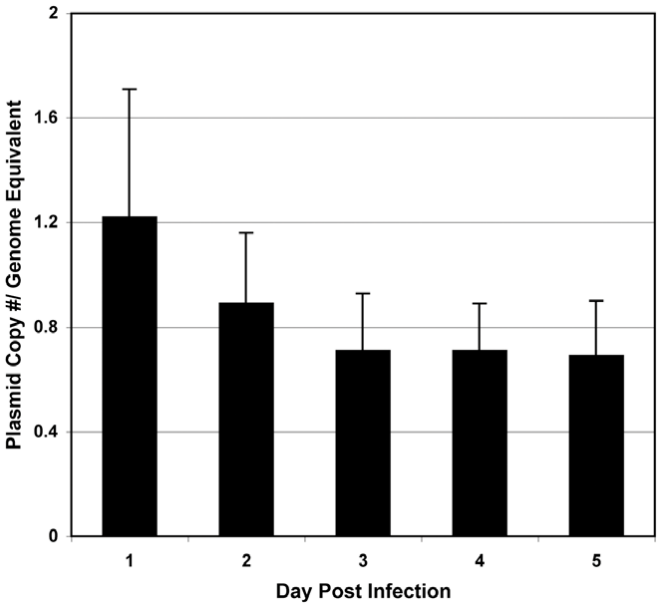
Plasmid copy number determination. Plasmid copy number/rickettsial genome equivalent was determined by comparing genome equivalents of the rickettsial chromosomal gene, *rho*, and the plasmid-borne *gfp_uv_* gene. Genome equivalents were determined by comparison to standard curves. Two independent biological samples were analyzed in duplicate.

Plasmid copy number was determined using QPCR. The relative ratio of the plasmid *gfp_uv_* gene to the single-copy *R. prowazekii rho* chromosomal gene was determined using gene-specific primers (Table 1). Copy number was evaluated over two growth curves at daily intervals for five days (10 independent determinations). These experiments revealed (Fig. 4) that pRAM18dRGA maintained a low copy number per rickettsia of approximately 1 (0.86+/−0.3, mean+/−S.D.) which falls at the lower range of rickettsial plasmid copy numbers (2.4–9.2) established for naturally-occurring rickettsial plasmids [11].

## Discussion

This study documents for the first time that *R. prowazekii* can support plasmid replication. Characterization of rickettsial plasmids identified in non-pathogenic rickettsial species led to the construction of the vector pRAM18dRGA [15]. This plasmid contains a 7.3 kb fragment from the replicative pRAM18 plasmid of *R. amblyommii* [11] ligated to a pGEM vector, containing the *rpsL^P^-Rparr-2/ompA^P^-gfp_uv_* selection/detection cassette [6], for replication in *E. coli*. This vector represents the smallest pRAM18 derived plasmid tested that was capable of replicating in a non-*R. amblyommii* rickettsial species and encodes proteins with homology to the DnaA-like replication initiator and the ParA plasmid partitioning protein [15].

pRAM18dRGA, was introduced into pathogenic *R. prowazekii* via electroporation and transformants isolated using rifampin selection. The observation of GFP_uv_ expression, under the control of a rickettsial promoter, also demonstrates that plasmid-borne genes can be expressed and detected, providing a promising first step in complementation assays. Although pRAM18dRGA was maintained at a low copy number, this is characteristic of known rickettsial plasmids and suggests that the machinery for maintaining plasmids in this pathogenic species is functional. Interestingly, when this plasmid was transformed into several other rickettsial species, the copy number of pRAM18dRGA was noticeably higher, ranging from 5.5+/−0.65 to 28.1+/−1.89 [15]. The low copy number of pRAM18dRGA in *R. prowazekii* supports its future use in expression studies, alleviating concerns of over-expression of multi-copy plasmid-borne genes in complementation assays.

The *R. amblyommii* fragment in pRAM18dRGA encodes only four proteins; a DnaA-like protein, a ParA partitioning protein, a TPR repeat-containing protein, and a homolog to Xre, a putative repressor protein. The DnaA-like protein was initially described during the annotation of the pRF plasmid of *R. felis* [10]. Interestingly, the annotation is based on homology of the carboxy-terminal 75 amino acids of the plasmid-expressed protein with the first 65 amino acids of the archetypical DnaA protein of *E. coli* [19]. The N-terminal region, or Domain I, of the DnaA protein is responsible for the loading of the helicase, DnaB [20]. However, the remaining 700 amino acids of the DnaA-like protein show no homology to DnaA and display no conserved domains, despite their conservation among the rickettsial plasmid sequences. In contrast to the highly conserved rickettsial chromosomal *parA* genes, the *parA* genes from several rickettsial plasmids were found to be highly diverse and clustered with *parA* genes found on plasmids from other bacterial genera [11], [21]. In addition to these DnaA-like and ParA proteins, the pRAM fragment encodes a protein with a tetratricopeptide repeat (TPR) motif, originally identified in yeast as a protein-protein interaction module, and a protein with homology to a helix-turn-helix transcription regulator, Xre. Interestingly, the Xre homolog in *Bacillus subtilis* is described as a probable repressor necessary for the maintenance of the lysogenic state of the defective prophage pbsX [22].

Fortunately, for future studies that might employ pRAM18dRGA, the presence of a plasmid appeared to have minimal effects on *R. prowazekii* growth. Growth of the plasmid-containing strain was comparable to that of wild-type Madrid E strain. In fact the plasmid-containing strain exhibited a slightly shorter generation time than the wild-type control. However, in the growth experiments presented here, the control Madrid E bacteria was isolated from hen egg yolk sacs and only passaged in L929 cells for two days prior to initiation of growth curve assays. A recent report demonstrated that the Madrid E strain grows slower in cell culture without adaptation to the specific host cell environment [23]. However, the 13-hour replication time is similar to the published 8–12 hour generation time [24], [25], [26], [27].

The demonstration of plasmid replication in *R. prowazekii* provides an important genetic tool and model genetic system for the study of this obligate intracellular pathogen. The absence of an extrachromosomal platform for the genetic analysis of *R. prowazekii* has prevented the evaluation of gene function by classical genetic complementation techniques. While it is possible to use transposon systems to evaluate gene complementation [8], an extrachromosomal location may be preferred for some experiments, since the plasmid would not disrupt the bacterial chromosome potentially contributing to an observed phenotype. The maintenance of the pRAM18dRGA shuttle plasmid suggests that other rickettsial plasmids may be maintained as well expanding the genetic tools available for the study of this rickettsial pathogen.
